# A prediction model to predict in-hospital mortality in patients with acute type B aortic dissection

**DOI:** 10.1186/s12872-023-03260-5

**Published:** 2023-05-17

**Authors:** Meng-meng Wang, Min-Tao Gai, Bao-zhu Wang, Gulinazi Yesitayi, Yi-Tong Ma, Xiang Ma

**Affiliations:** 1 Department of Cardiology, First Affiliated Hospital of Xinjiang Medical University, Urumqi, China; 2 Xinjiang Key Laboratory of Cardiovascular Disease, Clinical Medical Research Institute, First Affiliated Hospital of Xinjiang Medical University, Urumqi, 830011 China; 3 Xinjiang Key Laboratory of Medical Animal Model Research, Urumqi, 830011 China

**Keywords:** Acute type B aortic dissection, In-hospital mortality, Risk factors, Prediction model

## Abstract

**Background:**

Acute type B aortic dissection (ABAD) is a life-threatening cardiovascular disease. A practicable and effective prediction model to predict and evaluate the risk of in-hospital death for ABAD is required. The present study aimed to construct a prediction model to predict the risk of in-hospital death in ABAD patients.

**Methods:**

A total of 715 patients with ABAD were recruited in the first affiliated hospital of Xinjiang medical university from April 2012 to May 2021. The information on the demographic and clinical characteristics of all subjects was collected. The logistic regression analysis, receiver operating characteristic (ROC) curve analysis, and nomogram were applied to screen the appropriate predictors and to establish a prediction model for the risk of in-hospital mortality in ABAD. The receiver operator characteristic curve and calibration plot were applied to validate the performance of the prediction model.

**Results:**

Of 53 (7.41%) subjects occurred in-hospital death in 715 ABAD patients. The variables including diastolic blood pressure (DBP), platelets, heart rate, neutrophil-lymphocyte ratio, D-dimer, C-reactive protein (CRP), white blood cell (WBC), hemoglobin, lactate dehydrogenase (LDH), procalcitonin, and left ventricular ejection fraction (LVEF) were shown a significant difference between the in-hospital death group and the in-hospital survival group (all *P* < 0.05). Furthermore, all these factors which existed differences, except CRP, were associated with in-hospital deaths in ABAD patients (all *P* < 0.05). Then, parameters containing LVEF, WBC, hemoglobin, LDH, and procalcitonin were identified as independent risk factors for in-hospital deaths in ABAD patients by adjusting compound variables (all *P* < 0.05). In addition, these independent factors were qualified as predictors to build a prediction model (AUC > 0.5, *P* < 0.05). The prediction model was shown a favorable discriminative ability (C index = 0.745) and demonstrated good consistency.

**Conclusions:**

The novel prediction model combined with WBC, hemoglobin, LDH, procalcitonin, and LVEF, was a practicable and valuable tool to predict in-hospital deaths in ABAD patients.

## Introduction

Acute aortic dissection (AAD) is a serious rapidly progressive and life-threatening cardiovascular disease. Acute type B aortic dissection (ABAD), one type of AAD, is characterized as aortic dissections only involving the descending aorta and is accounting for 25–40% of all AAD [1]. The AAD disease usually develops promptly from the presentation of symptoms to the occurrence of life-threaten complications or even death. If appropriate treatments are not undertaken in time, the mortality of AAD will increase by 1–3% per hour after symptom presentation and exceed 50% within one week [2, 3]. Approximate 20% of patients with AAD died before hospitalization, and almost 30% died during hospitalization [4]. The 30-day mortality for ABAD was 13.3% [5]. The survival rate of AAD increased to 70% when early treatments were performed, such as aortic-repair surgery [6]. Therefore, a convenient measurement to predict and evaluate the risk of mortality immediately in patients with ABAD would be a valuable approach to restraining the development of ABAD and reducing mortality.

Recently, several studies have revealed the risk factors for AAD, such as sex, age, high blood pressure, smoking, dyslipidemia, and cocaine, et al. [7, 8]. Yet most studies had a small sample size. In addition, few prediction models were developed for AAD, especially for ABAD [9–11]. Because of the various clinical features in type A and type B aortic dissections for their potential different mechanisms, the prediction model would be built according to their own characteristics. Therefore, establishing a prediction model with a relatively large sample size is needed. Here, the present study enrolled 715 subjects, and their data and outcomes of in-hospital death or survival were invested. The predictors were identified in many potential factors, and a novel prediction model was established to predict and evaluate the risk of in-hospital deaths in ABAD patients. The prediction model would provide a convenient tool to assess the risk of in-hospital mortality in ABAD patients.

## Methods

### Participants

The subjects diagnosed with ABAD and according to the inclusion and exclusion criteria were recruited from the First Affiliated Hospital of Xinjiang Medical University between April 2012 to May 2021. The inclusion criteria were set as (1) age > 18 years; and (2) aortic dissection confirmed by computed tomography angiography (CTA). Exclusion criteria: (1) Stanford type A aortic dissection patients; (2) patients with acute myocardial infarction or acute cerebrovascular disease; (3) patients who had severe liver and kidney function diseases, malignant tumors, blood system diseases, or autoimmune diseases; and (4) patients diagnosed with hereditary connective tissue diseases such as Marfan syndrome.

**Fig. 1:**
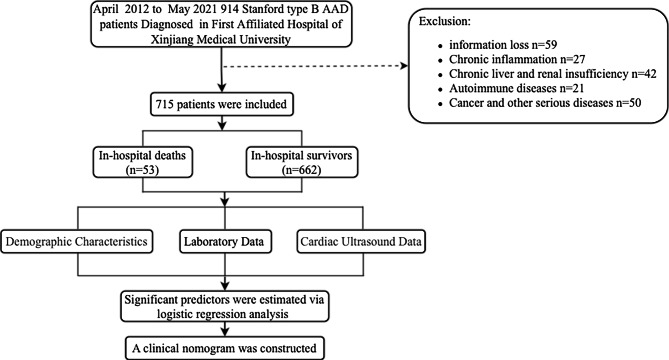
The study flowchart of the procedure of recruiting eligible subjects

A total of 914 patients were diagnosed with ABAD at the First Affiliated Hospital of Xinjiang Medical University between April 2012 and May 2021. Of 199 cases were excluded according to the exclusion criteria: 59 patients had incomplete information, 27 patients had chronic inflammation, 42 patients had chronic liver and renal insufficiency, 21 patients had autoimmune diseases, and 50 patients had cancer or other serious diseases. At last, 715 subjects were enrolled in this study. The flow diagram is shown in Fig. 1.

### Diagnostic criteria

The diagnosis of ABAD was followed the guideline [12] that the primary rupture location and extent of involvement only involve the descending thoracic aorta and its distal part. If the first onset of aortic dissection (AD) symptoms occurred within 14 days, it was classified as AAD. The subjects who had a history of hypertension, taking antihypertensive medication, or detected resting blood pressures larger than 140/90 mmHg on at least two separate occasions were defined as Hypertension [6]. The criteria to diagnose diabetes mellitus was set as the following three criteria: (1) fasting plasma glucose (FPG) levels ≥ 7.0mmol/L; (2) 2-hour plasma glucose ≥ 11.1 mmol/L during OGTT (75 g anhydrous glucose); (3) HbA1c ≥ 6.5% and occurring symptoms of hyperglycemia, and a random plasma glucose ≥ 11.1 mmol/L [13].

### Data collection

The data of demographic information, biochemical parameters, and clinical characteristics were collected for all participants. The demographic information contained age, sex, body weight, and height. Biochemical parameters included white blood cell (WBC), hemoglobin, platelets (PLT), creatinine (Cr), neutrophil-lymphocyte ratio (NLR), uric acid (UA), glucose (GLU), triglycerides (TG), total cholesterol (TC), low-density lipoprotein cholesterol (LDL-C), high-density lipoprotein cholesterol (HDL-C), lactate dehydrogenase (LDH), D-dimer, procalcitonin, interleukin 6 (IL-6), C-reactive protein (CRP). The clinical characteristics mainly recorded the parameters of heart rate (HR), systolic blood pressure (SBP), diastolic blood pressure (DBP), left ventricular ejection fractions (LVEF), history of smoking, drinking, hypertension, diabetes, and coronary heart disease (CHD).

### Statistical analysis

The SPSS 26.0 (SPSS Inc., Chicago, IL, USA) was applied to perform statistical analysis. Continuous variables were indicated as mean ± standard deviation (SDs) or medians and interquartile ranges (IQRs) depending on whether it is according to a normal distribution, and they were analyzed by unpaired Student’s *t*-test and Mann-Whitney *U*-test, respectively. Categorical variables were presented as percentages and analyzed with the Chi-square (χ^2^) test. All *P* values were two-tailed, and when *P* < 0.05 was considered statistically significant. A univariate logistic regression analysis was used to identify factors associated with in-hospital deaths in ABAD patients. Multivariate logistic regression analyses were conducted with the forward stepwise condition to select independent predictors and prediction models, of which included and excluded criteria were set as P-values <0.05 and >0.10, respectively. Then a receiver operator characteristic curve (ROC) method was applied to screen the eligible factors for the predicted model (area under curve > 0.5, *P* < 0.05). In further, a predictive nomogram was constructed based on statistical significance with R software version 4.0 (R Foundation for Statistical Computing, Vienna, Austria). The Hosmer–Lemeshow χ^2^ statistic, calibration curve, and internal validation by bootstrap repetitions 1000 times were used to evaluate the prediction model.

## Results

### Baseline characteristics of participants

A total of 715 ABAD patients were included in this study, of 53 (7.41%) occurred in-hospital deaths. As Table 1 demonstrated, the baseline characteristics were comparably in most parameters between the in-hospital death group and the in-hospital survival group in ABAD patients, which included age, sex, history of smoking, drinking, hypertension, CHD, diabetes mellitus (DM), SBP, Cr, UA, GLU, TG, TC, HDL-C, and LDL-C (all *P*>0.05). The in-hospital death group showed significantly lower levels of DBP, LVEF, hemoglobin, PLT, and higher HR, WBC, NLR, LDH, D-dimer, procalcitonin, and CRP than the in-hospital survival group (all *P* < 0.05).

**Table 1:** The comparations of baseline characteristics between the in-hospital survival and the in-hospital death groups in patients with ABAD.

Variables	In-hospital survivors (n = 662)	In-hospital deaths (n = 53)	T/χ^2^/*Z*	*P* value
Age(years)	51.97 ± 11.62	53.45 ± 13.40	-0.884	0.377
Male (n, %)	537(81.10)	45(84.90)	0.465	0.495
Smoking (n, %)	317(47.90)	25(47.20)	0.010	0.920
Drinking (n, %)	191(28.90)	16(30.20)	0.043	0.836
Hypertension (n, %)	488(73.70)	36(67.90)	0.841	0.359
CHD (n, %)	61(9.20)	6(11.30)	0.256	0.613
DM (n, %)	41(6.20)	3(5.70)	0.024	0.877
SBP (mmHg)	149.10 ± 27.64	142.21 ± 29.85	1.736	0.083
DBP (mmHg)	84.60 ± 17.13	79.30 ± 20.23	2.136	0.033^*^
Heart rate (/min)	82.74 ± 15.61	88.45 ± 19.33	-2.504	0.012^*^
LVEF (%)	61.26 ± 5.53	59.26 ± 4.39	2.566	0.010^*^
WBC(×10^9^/L)	11.05 ± 4.01	13.57 ± 5.48	-3.272	0.002^*^
NLR	6.53(3.69,10.87)	8.69(5.29,14.49)	-2.767	0.006^*^
Hemoglobin (g/L)	133.63 ± 21.26	125.79 ± 25.68	2.538	0.011^*^
Platelets (×10^9^/L)	215.74 ± 95.44	185.64 ± 73.91	2.784	0.007^*^
Creatinine (µmol/L)	78.32(65.82,100.46)	85.29(63.77,157.60)	-1.291	0.197
Uric acid (µmol/L)	339.50 ± 129.53	370.03 ± 168.40	-1.290	0.202
Glucose (mmol/L)	7.07 ± 2.44	7.25 ± 2.04	-0.521	0.603
TG (mmol/L)	1.59 ± 1.01	1.62 ± 0.79	-0.243	0.808
TC (mmol/L)	3.94 ± 0.93	3.84 ± 0.86	0.774	0.439
HDL-C(mmol/L)	0.98 ± 0.31	0.93 ± 0.30	0.923	0.287
LDL-C(mmol/L)	2.45 ± 0.73	2.30 ± 0.70	1.424	0.155
LDH [U/L, *M (Q*_*1*_, *Q*_*3*_*)*]	179.76(144.22,231.07)	273.36(158.62,462.37)	-3.966	< 0.001^*^
D-dimer (ng/mL)	1018.00(647.00,1827.50)	2344.00(1010.50,3014.50)	-4.407	< 0.001^*^
Procalcitonin (ng/mL)	0.08(0.05,0.18)	0.34(0.06,0.82)	-4.208	< 0.001^*^
CRP (mg/L)	16.55(6.25,44.31)	44.56(21.72,90.00)	-4.089	< 0.001^*^

^*^P < 0.05, compared with the In-hospital survival group. Abbreviations: ABAD, Acute type B aortic dissection; CHD, coronary heart disease; DM, diabetes mellitus; SBP, systolic blood pressure; DBP, diastolic blood pressure; LVEF, left ventricular ejection fraction; WBC, white blood cell; NLR, neutrophil-lymphocyte ratio; TG, triglycerides; TC, total cholesterol; HDL-C, high density lipoprotein cholesterol; LDL-C, low density lipoprotein cholesterol; LDH, lactate dehydrogenase; CRP, C-reactive protein

### Identifying potential predictors

To obtain potential predictors for in-hospital deaths in ABAD patients, univariate and multivariate logistic analyses were applied. The parameters that contained DBP, platelets, heart rate, NLR, D-dimer, WBC, hemoglobin, LDH, procalcitonin, and LVEF were been proven to be associated with in-hospital deaths in ABAD patients (all *P* < 0.05), and their specific values were indicated in Table 2. In further, these variables including WBC, hemoglobin, LDH, procalcitonin, and LVEF were also identified as independent predictors for in-hospital deaths in ABAD patients by adjusting compound factors included DBP, HR, NLR, PLT, D-dimer, and CRP, and their values of ORs and 95% CIs were LVEF (OR: 0.938; 95% CI: 0.895 to 0.982; *P* = 0.006), WBC (OR: 1.144; 95% CI: 1.072 to 1.221; *P* < 0.001), hemoglobin (OR: 0.983; 95% CI: 0.970 to 0.995; *P* = 0.007), LDH (OR: 1.000; 95% CI: 1.000 to 1.001; *P* = 0.043), and procalcitonin (OR: 1.084; 95% CI:1.007 to 1.168; *P* = 0.033), respectively.

**Table 2:** Logistic regression analysis of the risk factors for in-hospital death in ABAD patients

Variables	Univariate			Multivariate	
	OR (95% CI)	P value		OR (95% CI)	*P* value
DBP (mmHg)	0.981(0.964 to 0.998)	0.032^*^			
Heart rate (/min)	1.021(1.004 to 1.038)	0.013^*^			
LVEF (%)	0.947(0.908 to 0.988)	0.012^*^	0.938(0.895 to 0.982)		0.006^*^
WBC(×10^9^/L)	1.134(1.067 to 1.204)	< 0.001^*^	1.144(1.072 to 1.221)		< 0.001^*^
NLR	1.035(1.008 to 1.062)	0.009^*^			
Hemoglobin (g/L)	0.984(0.972 to 0.996)	0.012^*^	0.983(0.970 to 0.995)		0.007^*^
Platelets (×10^9^/L)	0.996(0.992 to 0.999)	0.023^*^			
LDH (U/L)	1.000(1.000 to 1.001)	0.001^*^	1.000(1.000 to 1.001)		0.043^*^
D-dimer (ng/mL)	1.000(1.000 to 1.000)	0.029^*^			
Procalcitonin (ng/mL)	1.131(1.041 to 1.228)	0.004^*^	1.084(1.007 to 1.168)		0.033^*^
CRP (mg/L)	1.003(0.999 to 1.008)	0.139			

^*^P < 0.05. Abbreviations: ABAD, Acute type B aortic dissection; DBP, diastolic blood pressure; LVEF, left ventricular ejection fraction; WBC, white blood cell; NLR, neutrophil-lymphocyte ratio; LDH, lactate dehydrogenase; CRP, C-reactive protein

### Screening predictors to construct a prediction model

ROC analysis was conducted to identify eligible predictors to include in the prediction model for in-hospital deaths in ABAD patients. As Table 3 shown, the variables that were independent predictors were also entered the prediction model, their AUCs and 95% CIs were WBC: 0.635 (95% CI: 0.551–0.719), hemoglobin: 0.589 (95% CI: 0.504–0.673), LDH 0.664 (95% CI: 0.576–0.752), procalcitonin: 0.673 (95% CI: 0.587–0.759), LVEF: 0.652(95% CI: 0.584–0.720), respectively, all *P* < 0.05.

**Table 3:** ROC analysis for each predictor of in-hospital death in ABAD patients

Variables	Sensitivity (%)	Specificity (%)	AUC	95%CI	*P* value
WBC	49.10	75.20	0.635	0.551–0.719	0.001^*^
Hemoglobin	56.60	62.30	0.589	0.504–0.673	0.031^*^
LDH	50.90	84.30	0.664	0.576–0.752	< 0.001^*^
Procalcitonin	54.70	82.00	0.673	0.587–0.759	< 0.001^*^
LVEF	66.00	64.20	0.652	0.584–0.720	< 0.001^*^
Combine	60.40	81.30	0.745	0.674–0.815	< 0.001^*^

^*^P < 0.05. Abbreviations: ABAD, Acute type B aortic dissection; WBC, white blood cell; LDH, lactate dehydrogenase; LVEF, left ventricular ejection fraction

### Construction of nomogram

**Fig. 2:**
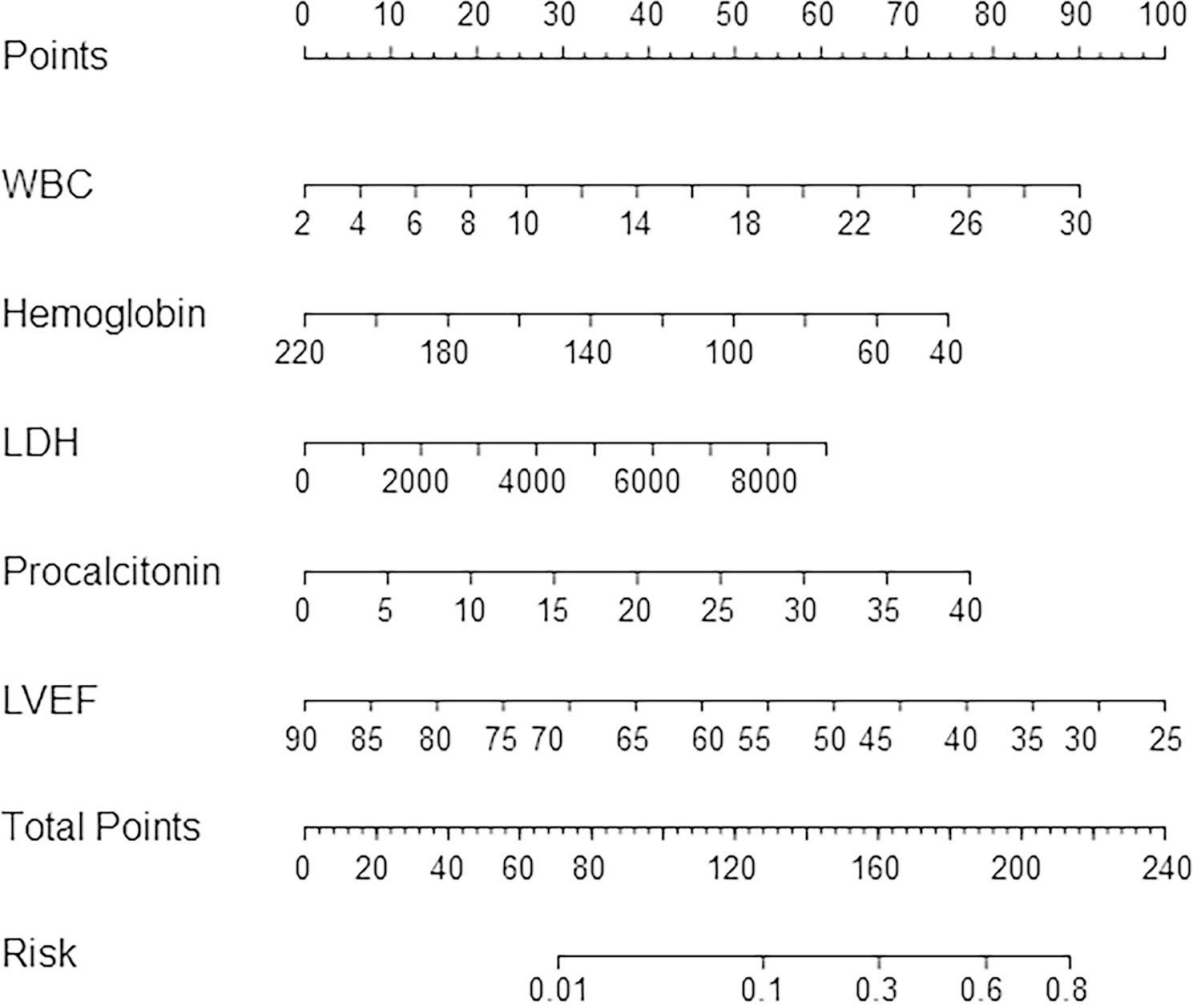
**A nomogram for the prediction model** *Using instruction*: a line labeled “Points” is a corresponding score for each variable. The line labeled “Total points” suggests total points which were evaluated by all variables in the model for an individual, and it corresponds to the line marked “Risk” which represents the risk of in-hospital death for patients with ABAD. *Abbreviations*: ABAD, Acute type B aortic dissection; WBC, white blood cell; LDH, lactate dehydrogenase; LVEF, left ventricular ejection fraction

Establishing a nomogram could contribute to the application of the predictive model in clinics. The nomogram, used to quantitatively predict the risk of in-hospital deaths in ABAD patients, was constructed (Fig. 2). The variables, including WBC, hemoglobin, LDH, procalcitonin, and LVEF were configured. The total score can be counted for each patient by evaluating the score of five variables. The patients indicated higher total scores, they are probably accompanied by a higher risk of in-hospital deaths.

### The performance of the nomogram

**Fig. 3:**
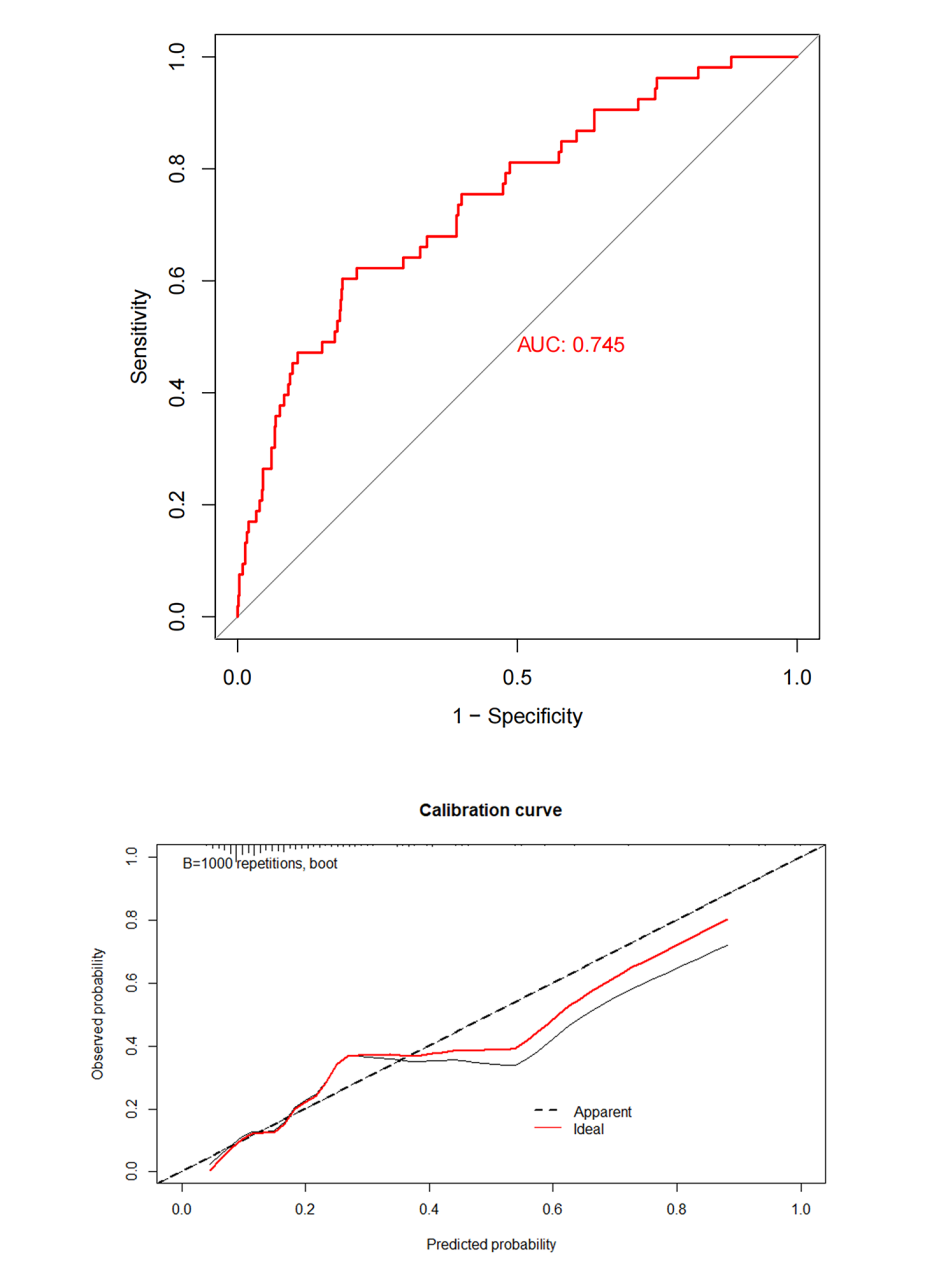
**Using C-index analysis and calibration curve to evaluate the prediction model.** (**A**) The C-index analysis was performed. (**B**) The calibration curve displayed the deviation between predicted and observed probability. Repetitions 1000 times were performed for internal validation

A C-index was used to assess the discriminatory capacity of the model to detect the risk of in-hospital deaths in ABAD patients, which is presented in Fig. 3A. The C-index, also expressed as AUC, was 0.745, which indicated a high capability of the model to distinguish patients with or without a high risk of in-hospital deaths. To evaluate the predictive model, Hosmer–Lemeshow goodness-of-fit test and calibration curve were performed. The *P* value of the Hosmer–Lemeshow goodness-of-fit test was 0.481, shown no deviation. In addition, the calibration curve with an internal validation of 1000 times repetitions was shown in Fig. 3B, indicating no deviation and good consistency between predicted and observed probability.

## Discussion

Here, we built a prediction model by screening the parameters which were commonly examined in clinics to evaluate the risk of in-hospital deaths. In the present study, the in-hospital mortality in ABAD patients was 7.41%, and the parameters of WBC, hemoglobin, LDH, procalcitonin, and LVEF were independent predictors for in-hospital deaths in ABAD patients. Further, a novel prediction model and nomogram were constructed by these predictors. The prediction model may provide a tool for clinicians to perform risk stratification and to prevent in-hospital death in potential ABAD patients.

The in-hospital mortality of ABAD was 7.41% in the current study which included 715 subjects. A report from the international registry of acute aortic dissection (IRAD), which investigated 1034 AAD patients between 1996 and 2013, showed the in-hospital mortality rate was 10.6% [10]. A survey between 2003 and 2011 in 250 ABAD patients also showed a 10% in-hospital mortality [14]. In addition, there were two separate studies in 384 and 166 ABAD patients both indicated their overall in-hospital mortality was around 13% [15, 16]. Compared with the previously reported 9-13% mortality of ABAD, the mortality in our study was a little lower [9, 16, 17], which may attribute to the progression of medical technology in the recent decade, for our research was performed from 2012 to 2021. We also noticed that few data about pre-hospitalization mortality of ABAD were reported. Unfortunately, the data was not obtained in this study because the pre-hospitalization death patients were not included in the present study and its data was hard to collect.

The parameters including WBC, hemoglobin, LDH, procalcitonin, and LVEF were used to establish the prediction model in the present study, and some of the parameters have not been reported as risk factors for ABAD before. We were aware of the clinical variables and classifications in previous studies provided very important references for clinicians to evaluate ABAD. These clinical variables include maximum aortic diameter [18], dilatation of the thoracic aorta [19], uncontrolled hypertension [20], hypotension/shock, absence of chest/back pain on presentation, and branch vessel [15]. Recently, the Penn classification was also been proven to be practicable in predicting ABAD in-hospital death [16]. In addition, some routine indicators like age, sex, WBC, NT-proBNP, and platelet to lymphocyte ratio have also been reported that they were associated with in-hospital deaths in ABAD [9, 21]. Although clinical variables related to aortic were more effective to diagnose ABAD, we are more prone to build a prediction model by screening the parameters which were commonly examined in clinics to enhance the practicability of this prediction model in ordinary people to find potential ABAD patients earlier. In addition, many studies indicated potential predictors and risk factors FOR ABAD patients, but few prediction models were constructed. Furthermore, most of these factors associated with ABAD were evaluated in relatively small sample sizes.

Lately, there was a study building a prediction model based on 188 ABAD patients, which included the variables of hypotension, ischemic complications, renal dysfunction, and neutrophil percentage [17]. Similar to the published study, the parameters related to inflammation were contained in the prediction model in our study. However, although the parameter of DBP was also lower in ABAD in-hospital death group in our study, it was not an independent predictor for ABAD. But compared with this published study, the prediction model in our study identified more predictors with a much larger sample size, and was evaluated and validated internally.

The parameter of WBC has been reported before. The WBC is a sensitive inflammatory marker. Several studies manifested that elevated WBC might be used as a predictor for increased risk of in-hospital deaths in type A aortic dissection patients [22] and ABAD patients [23]. Previous studies indicated systemic inflammatory response acted as a pivotal role and was early onset in the development of AAD [24, 25]. The infiltration of multiple immune and inflammatory cells was the primary pathological feature for patients with AAD because it promotes elastic fiber degradation and aortic wall medial degeneration [26]. Similar to WBC, procalcitonin was also one of the independent predictors in the prediction model after adjusting confounding factors including CRP et al. As a procalcitonin precursor protein, procalcitonin is produced by monocytes and hepatocytes, and it reflects the activity of the systemic inflammatory response. procalcitonin could predict postoperative outcomes and mortality in the AAD population [27–29], but whether it exerts similar effects on ABAD patients was not revealed before. Therefore, both WBC and procalcitonin would be important predictors to construct the prediction model for they are early occurred biomarkers for the inflammatory response in ABAD patients.

In the current prediction model, LDH was also an independent predictor included in the model. LDH is a key enzyme in the glycolysis pathway to catalyze the redox reaction between pyruvate and lactate. One study measured the concentration of tissue lactate in aorta samples of AD patients, which showed elevated lactate [30]. Excessive LDH could enhance lactate synthesis in vascular smooth muscle cells (VSMCs) [31] and affect the phenotypic transformation of VSMCs and the expression of MMP2/9, which leads to the development of aortic dissection. He et al. showed that LDH was positively correlated with AAD patients’ in-hospital mortality [32]. Yet the knowledge of LDH played in ABAD was limited. Our results identified that a high LDH level was an independent predictor for in-hospital mortality in ABAD patients, which probably attributed to the higher LDH-induced phenotypic transformation of VSMCs and the expression of MMP2/9.

Meanwhile, hemoglobin was one of the variables in the prediction model. Hemoglobin is an important oxygen carrier in the blood. In recent years, it has been enclosed that it was a prognostic marker for patients with cardiovascular diseases or undergoing cardiac surgery [33–35]. Consistent with the current study, recent studies showed that hemoglobin was not only associated with in-hospital events but also influenced long-term outcomes in ABAD patients who suffered thoracic endovascular aortic repair surgery [36, 37]. Moreover, the elevated hemoglobin is related to abdominal aortic aneurysm diameter and reduced long-term survival in patients undergoing endovascular abdominal aortic aneurysmal repair [38]. However, how hemoglobin interacted with ABAD is not clear. A basic animal study indicated the application of exogenous hemoglobin could attenuate aortic endothelial dysfunction [39], which implied that the increased level of hemoglobin would reduce mortality of ABAD by ameliorating aortic endothelial dysfunction.

LVEF was involved in the prediction model. The appropriate value of LVEF usually represented a healthy systolic cardiac function. Reduced LVEF was an independent predictor for mid-term mortality in AAD patients who underwent total arch replacement, and when the LVEF value is less than 55% will be recognized as a poor prognosis [40]. However, little evidence proved that LVEF was a predictor of in-hospital mortality in patients with ABAD. Here, we proved that LVEF demonstrated good predictive ability for the risk of in-hospital mortality in ABAD patients, which means that the selection of therapeutic strategies for patients with reduced LVEF should be careful.

Some limitations existed in the current study. Firstly, this was a retrospective-designed study, which may limit the scope of application for the prediction model. Secondly, the prediction model was only validated in internal repetition since it is difficult to find other cooperative institutes that have collected enough sample sizes to perform an external validation. Third, the follow-up period is relatively short, a long-term follow-up study is needed to further test the value of the prediction model in subacute and chronic stages of ABAD disease for these stages occupied a larger proportion of mortality.

In conclusion, a novel prediction model was developed by combining the routinely available clinical variables (WBC, hemoglobin, LDH, procalcitonin, and LVEF) to predict and evaluate the risk of in-hospital deaths in patients with ABAD. In addition, this study provided a convenient and practicable nomogram for clinicians to evaluate the risk of in-hospital deaths in ABAD patients.

## Data Availability

The datasets used and analysed during the current study available from the corresponding author on reasonable request.

## List of Abbreviations

AAD: Acute aortic dissection
ABAD: Acute type B aortic dissection
CTA: Computed tomography angiography
FPG: Fasting plasma glucose
WBC: White blood cell
NLR: Neutrophil-lymphocyte ratio
PLT: Platelets
Cr: Creatinine
UA: Uric acid
GLU: Glucose
TG: Triglycerides
TC: Total cholesterol
LDL-C: Low-density lipoprotein cholesterol
HDL-C: High-density lipoprotein cholesterol
LDH: Lactate dehydrogenase
IL-6: Interleukin 6
CRP: C-reactive protein
LVEF: Left ventricular ejection fraction
HR: Heart rate
SBP: Systolic blood pressure
DBP: Diastolic blood pressure
CHD: Coronary heart disease
DM: Diabetes mellitus
VSMCs: Vascular smooth muscle cells
